# Safety and Efficacy of Deep Venous Thrombosis Chemoprophylaxis in Neurosurgery Patients

**DOI:** 10.7759/cureus.115544

**Published:** 2026-08-31

**Authors:** Neha Philip, Anthony Nguyen, Awais Z Vance

**Affiliations:** 1 Neurosurgery, Baylor Scott & White Medical Center - Temple, Temple, USA

**Keywords:** bleeding complications, deep vein thrombosis (dvt), deep venous thrombosis (dvt), dvt chemoprophylaxis, postoperative neurosurgical patients, pulmonary embolism (pe)

## Abstract

Background and objective: Deep venous thrombosis (DVT) and pulmonary embolism (PE) remain significant sources of morbidity and mortality in neurosurgical patients. Although chemoprophylaxis has been shown to reduce thromboembolic events, its routine use is controversial due to the potential risk of hemorrhagic complications. This study evaluated the safety and efficacy of DVT chemoprophylaxis compared with no chemoprophylaxis in postoperative neurosurgical patients.

Methods: We conducted a single-center retrospective chart review of adult patients who underwent neurosurgical procedures in 2019 with a hospital stay ≥5 days. The primary outcome was the incidence of symptomatic venous thromboembolism (VTE) within 90 days. Secondary outcomes measured were bleeding, wound complications, and return to the operating room within 30 days. Multivariable logistic regression was performed to assess the association of chemoprophylaxis with outcomes, adjusting for key covariates. Subgroup analysis was conducted to evaluate the effect of prophylaxis timing.

Results: A total of 340 patients met the study criteria. Chemoprophylaxis was administered to 176 patients (51.8%). Symptomatic VTE occurred in seven patients (2.06%). Use of chemoprophylaxis was not associated with reduced VTE risk (OR 4.6, p = 0.17). Spinal surgery compared to cranial surgery (OR 13.7, p = 0.03) and prior VTE history (OR 26.0, p = 0.03) were independently associated with higher VTE risk. Bleeding and wound complications were rare (<1%) and were not associated with chemoprophylaxis. Subgroup analysis of patients who received chemoprophylaxis demonstrated that timing of initiation was not significantly associated with VTE rates.

Conclusion: No statistically significant association was detected between DVT chemoprophylaxis and symptomatic VTE in this cohort. The higher observed VTE rate among patients receiving chemoprophylaxis should not be interpreted as evidence of reduced efficacy or increased VTE risk given the non-randomized design and potential for confounding by indication. The low frequency of bleeding and wound complications is reassuring, but the small number of events limits conclusions regarding safety. Individualized risk stratification may be particularly important for patients undergoing spinal surgery or with prior VTE. Prospective multicenter studies are needed to better define optimal prophylaxis strategies.

## Introduction

Deep venous thrombosis (DVT) and pulmonary embolism (PE) are significant causes of morbidity and mortality in neurosurgical patients. The risk of venous thromboembolism (VTE) is increased by factors common to this population, including prolonged surgery, postoperative immobility, and advanced age [1]. Reported rates of DVT among neurosurgical patients range from 7.4% to 15.3% [1-4], highlighting the importance of effective postoperative prophylaxis.

Chemoprophylaxis can reduce the risk of postoperative DVT and PE, but its use must be balanced against the potential for hemorrhagic complications, which may be particularly serious following neurosurgical procedures [5-12]. Although prior studies have demonstrated a reduction in VTE with pharmacologic prophylaxis, the literature remains mixed regarding its safety and optimal use. Current guidelines similarly differ in their recommendations, with some favoring mechanical prophylaxis alone and others supporting the addition of chemoprophylaxis in patients at higher risk of VTE once adequate hemostasis has been achieved [13,14]. Thus, uncertainty remains regarding the safety and effectiveness of chemoprophylaxis in routine postoperative neurosurgical care.

The primary objective of this retrospective, non-randomized study is to compare the safety and VTE outcomes associated with postoperative DVT chemoprophylaxis versus no chemoprophylaxis in neurosurgical patients. We will evaluate differences in documented DVT/PE, hemorrhagic complications, and other postoperative complications. Given the retrospective design, these comparisons will assess associations rather than directly establish treatment efficacy while accounting for potential differences in baseline VTE risk between groups. The secondary objective is to evaluate the association between timing of chemoprophylaxis initiation and postoperative DVT/PE.

## Materials and methods

This is a retrospective chart review of all patients aged 18 years and older who underwent spine or cranial surgery in the operating room at our institution in 2019 and had a hospital length of stay of at least five days. This minimum length-of-stay criterion was chosen to mirror the cohort in a prior study by Byrne et al., which limited inclusion to patients who remained alive and hospitalized for at least five days following surgery [15]. Patients were identified through institutional surgical and hospital records and were categorized according to whether they received pharmacologic DVT prophylaxis during their hospitalization. Because prophylaxis was determined by the treating clinical team and was not randomly assigned, treatment groups were expected to differ in baseline VTE and bleeding risk. The study was reviewed and approved by the Baylor Scott & White Research Institutional Review Board (approval no. 024-105) and was conducted in accordance with their policies.

Measures

The primary outcome was symptomatic VTE, defined as objectively confirmed DVT or PE occurring within 90 days of the index neurosurgical procedure. Deep venous thrombosis was confirmed by duplex ultrasonography, and PE was confirmed by contrast-enhanced CT. Follow-up for VTE was obtained through review of available institutional records and documented encounters during the 90-day follow-up period. Secondary outcomes included surgical site bleeding, wound complications, and unplanned return to the operating room within 30 days. Surgical site bleeding was defined as symptomatic hemorrhage in or from the surgical site, including symptomatic epidural hematoma, continued drainage for >7 days, or bleeding requiring oversewing. Wound complications included wound dehiscence, wound infection, or continued drainage for >7 days.

Independent variables of interest included patient age, sex, medical comorbidities (hypertension, diabetes, chronic kidney disease, history of malignancy, and history of prior VTE), surgical site (cranial versus spinal), surgical urgency (elective versus emergent), and use of chemical DVT prophylaxis. Age was dichotomized as <50 or ≥50 years, based on prior research identifying this threshold as a significant predictor of lower extremity DVT in neurosurgical patients [16]. 

Pharmacologic DVT prophylaxis was recorded as a binary exposure variable. Patients who received any pharmacologic prophylaxis before or after surgery during the index hospitalization were classified as receiving chemoprophylaxis. The specific agent, dose, duration, and timing relative to surgery were recorded when available in the medical record. Prophylaxis was administered according to the treating neurosurgical team’s clinical judgment, based on perceived thromboembolic and bleeding risk and postoperative hemostasis. There was no standardized protocol requiring prophylaxis for all patients. Reasons for initiating or withholding prophylaxis were not consistently documented because of the retrospective nature of the study. Mechanical prophylaxis was also recorded when documented, including the use of intermittent pneumatic compression devices and/or compression stockings. Because documentation of mechanical prophylaxis was not uniform, its use could not be reliably incorporated into all analyses.

The timing of pharmacologic prophylaxis relative to surgery was categorized as preoperative or postoperative, with postoperative initiation recorded by postoperative day. Duration of prophylaxis was recorded when available. Patients receiving prophylaxis at different time points were included in the overall chemoprophylaxis group regardless of timing.

Statistical analysis

The R software version 4.6.0 (R Foundation for Statistical Computing, Vienna, AUT) was employed for all statistical analyses. To examine the correlation of the outcomes of interest with independent variables, multivariable logistic regression was utilized. Logistic regression was only performed for outcomes of interest with >1% incidence. Covariates were screened for inclusion in multivariable logistic regression by performing univariable logistic regression between the outcome of interest and each aforementioned independent variable. Any variable that met the a priori specified threshold of p <0.20 was included in the final multivariable logistic regression analysis. The coefficients of the variables were then transformed into odds ratios. An alpha of 0.05 was specified a priori.

A subgroup analysis examining whether the timing of DVT prophylaxis was associated with the incidence of symptomatic VTE was performed. As the timing of DVT prophylaxis would correlate significantly with whether patients received DVT prophylaxis and introduce multicollinearity to the multivariable logistic regression model, this analysis was conducted separately and included only patients who received chemical DVT prophylaxis. Fisher’s exact test was performed to compare the incidence of symptomatic VTE with various thresholds of timing of DVT prophylaxis. Two groups were formed for each analysis. Patients who were started on chemical DVT prophylaxis preoperatively or by postoperative day one constituted a group and were compared to patients who were started on DVT prophylaxis after postoperative day one. This was repeated with thresholds of postoperative days two to five.

Because chemoprophylaxis was not randomly assigned, the analyses were interpreted as estimates of association rather than treatment efficacy. In particular, the potential for confounding by indication was considered, as patients at greater perceived risk for VTE may have been more likely to receive prophylaxis. The small number of symptomatic VTE events also limited the number of covariates that could be reliably incorporated into multivariable models and resulted in wide confidence intervals. Accordingly, adjusted estimates were interpreted with caution and considered hypothesis-generating.

A subgroup analysis evaluated whether the timing of chemoprophylaxis was associated with symptomatic VTE among patients who received prophylaxis. Timing was analyzed separately because inclusion of timing and the binary chemoprophylaxis variable in the same model could introduce multicollinearity. Fisher’s exact test was used to compare symptomatic VTE rates according to different prophylaxis timing thresholds. For each analysis, patients initiated on chemoprophylaxis preoperatively or by postoperative day 1 were compared with those initiated after postoperative day 1. The analysis was repeated using thresholds of postoperative days two to five.

Missing data were assessed during chart review. Variables with incomplete documentation were analyzed using available-case data, and no values were imputed unless otherwise specified. Because prophylaxis indications, mechanical prophylaxis use, and certain treatment characteristics were not consistently documented, these variables were considered potential sources of residual confounding and limitations to interpretation.

## Results

A total of 340 patients underwent neurosurgical intervention during a hospital admission of at least five days' duration during the study period. Baseline demographic and patient data are presented in Table 1. Of these 340 patients, 176 were initiated on DVT chemoprophylaxis at any point during the hospitalization (51.8%), and there were seven patients with symptomatic VTEs (2.06%). The incidence of the outcomes of interest aside from symptomatic VTE was <1% each. Surgical site bleeding and wound complications each occurred in two patients (0.59%), and return to the operating room occurred in one patient (0.29%).

**Table 1:** Baseline study demographics broken down by whether patients received DVT chemical prophylaxis or not DVT: Deep venous thrombosis, VTE: Venous thromboembolism

Variable	Characteristics	Did not receive DVT chemoprophylaxis		Received DVT chemoprophylaxis		Total overall	
		N	Percentage	N	Percentage	N	Percentage
Age	< 50 years	40	24.39%	41	23.30%	81	23.82%
	> 50 years	124	75.61%	135	76.70%	259	76.18%
Sex	Male	83	50.61%	97	55.11%	180	52.94%
	Female	81	49.39%	79	44.89%	160	47.06%
Comorbidities	Hypertension	77	46.95%	95	53.98%	172	50.59%
	Diabetes mellitus	41	25.00%	51	28.98%	92	27.06%
	Chronic kidney disease	6	3.66%	15	8.52%	21	6.18%
	History of malignancy	11	6.71%	11	6.25%	22	6.47%
	Prior VTE	0	0.00%	4	2.27%	4	1.18%
Surgery site	Cranial	105	64.02%	105	60.80%	212	62.35%
	Spinal	59	35.98%	69	39.20%	128	37.65%
Elective surgery	Elective	56	34.15%	30	17.05%	86	25.29%
	Non-elective	108	65.85%	146	82.95%	254	74.71%
Outcomes	Incidence of DVT	1	0.61%	6	3.41%	7	2.06%
	Bleeding complication	1	0.61%	1	0.57%	2	0.59%
	Wound complication	2	1.22%	0	0.00%	2	0.59%
	Return to operating room	0	0.00%	1	0.57%	1	0.29%

Only three variables met univariable logistic screening thresholds: history of prior VTE, spinal versus cranial category, and utilization of DVT chemoprophylaxis. The p-values of univariable logistic analysis are presented in Table 2.

**Table 2:** The p-values of univariable logistic regression analysis of symptomatic VTE with the independent variables of interest. Variables with p-values of <0.20 were included in multivariable regression; VTE: Venous thromboembolism

Variable	OR (95% CI)	p-value
Age > 50 years	Infinity (undefined)	0.99
Sex	Infinity (undefined)	0.99
Hypertension	1.31 (0.28-6.73)	0.73
Diabetes mellitus	1.08 (0.15-5.11)	0.93
Chronic kidney disease	2.61 (0.13-16.32)	0.39
History of malignancy	2.48 (0.13-15.44)	0.41
Prior	18.33 (0.84-170.03)	0.02
Category (cranial versus spinal)	10.38 (1.74-197.20)	0.03
Elective surgery	1.19 (0.17-5.61)	0.84
Utilization of chemical prophylaxis	5.75 (0.97-109.28)	0.11

The multivariable logistic regression analysis including these three covariates in Table 3 demonstrated that only prior VTE (OR 26.0, 95% CI 0.9-796.8, p = 0.03) and spinal surgery as opposed to cranial surgery (OR 13.7, 95% CI 2.0-339.7, p = 0.03) were significantly associated with the incidence of symptomatic VTE. Regarding secondary outcomes, only chronic kidney disease met univariable logistic screening criteria for any of the outcomes. The p-value of chronic kidney disease in both the univariable models for bleeding complications and wound complications was 0.05, which did not meet statistical significance. Univariable analysis of return to the operating room did not reveal any covariates that met the screening threshold of p <0.20.

**Table 3:** Results of multivariable logistic regression of covariates meeting the univariable screening criteria A p-value <0.05 was considered significant; VTE: Venous thromboembolism

Variable		OR	95% CI	p-value
Prior VTE		26.0	0.9 - 796.8	0.03
Category	Cranial	Ref	Ref	Ref
	Spinal	13.7	2.0 - 339.7	0.03
Utilization of chemical prophylaxis		4.6	0.7 - 89.7	0.17

Among patients who received any DVT chemoprophylaxis, the timing of initiation was not significantly associated with the incidence of symptomatic VTE, as shown in Table 4. The lowest p-value produced by Fisher’s exact test examining prophylaxis timing was 0.40 for patients who were started on chemoprophylaxis on or before postoperative day two.

**Table 4:** Results of Fisher’s exact test analyzing timing of chemical DVT prophylaxis and incidence of symptomatic VTE among patients who received any chemical prophylaxis Comparison groups are those who were started on prophylaxis at later time points. DVT: Deep venous thrombosis; VTE: Venous thromboembolism

Timing of prophylaxis	OR	95% CI	p-value
Postoperative day #1 or sooner	0.38	0.01-3.49	0.67
Postoperative day #2 or sooner	0.28	0.01-2.58	0.40
Postoperative day #3 or sooner	0.83	0.11-6.37	1
Postoperative day #4 or sooner	0.53	0.07-4.11	0.43
Postoperative day #5 or sooner	0.66	0.09-7.52	0.64

## Discussion

Our findings add to the ongoing debate regarding the role of chemoprophylaxis in neurosurgical patients. In this retrospective cohort of 340 patients hospitalized for at least five days, DVT chemoprophylaxis was not significantly associated with a reduction in symptomatic VTE. Although patients receiving chemoprophylaxis had higher observed odds of symptomatic DVT (OR 4.6, 95% CI 0.7-89.7, p = .17), this finding should not be interpreted as evidence that chemoprophylaxis is ineffective. The higher observed VTE rate in the prophylaxis group may be influenced by confounding by indication, as patients perceived to be at higher risk for thromboembolism may have been more likely to receive chemoprophylaxis. Therefore, our findings demonstrate a lack of statistically significant association rather than evidence of a lack of treatment efficacy.

These findings differ from several prior studies demonstrating a reduction in DVT with pharmacologic prophylaxis. Dickinson et al. randomized patients undergoing craniotomy to preoperative low-molecular-weight heparin (LMWH) plus mechanical prophylaxis or mechanical prophylaxis alone; the study was terminated prematurely after five of 46 patients in the treatment group developed postoperative intracranial hemorrhage [9]. A systematic review by Collen et al. found that both LMWH and intermittent compression devices reduced DVT rates in neurosurgical patients, although LMWH was associated with higher rates of intracranial hemorrhage and minor bleeding [10]. In a multicenter, double-blind trial, Agnelli et al. reported DVT rates of 32% with placebo plus compression stockings compared with 17% with LMWH plus compression stockings, corresponding to a relative risk reduction of 52% without a statistically significant increase in adverse bleeding events [11]. Similarly, Alshehri et al. reported a 61% reduction in DVT with combined chemoprophylaxis and mechanical prophylaxis among patients undergoing craniotomy, with an increased risk of minor hemorrhage but no significant increase in major hemorrhage [12].

Differences in study design and patient populations likely contribute to the discrepancy between these findings and our results. Khaldi et al. reported a 43% reduction in lower-extremity DVT, while Alshehri et al. reported a 61% reduction [5,12]. However, these studies included screening ultrasound to identify asymptomatic DVT, whereas our study was limited to symptomatic VTE events. Alshehri et al. [12] also focused specifically on brain tumor patients undergoing craniotomy, while our cohort included a broader range of elective and urgent cranial and spinal procedures. The relatively low symptomatic VTE rate in our cohort, with only seven events among 340 patients, further limited the statistical power to detect a treatment effect. Thus, the absence of a statistically significant association in our study should be interpreted in the context of substantial statistical imprecision.

The identification of spinal surgery and prior VTE as factors associated with symptomatic DVT is consistent with previous literature [16]. Spinal surgery was associated with higher odds of symptomatic DVT compared with cranial surgery (OR 13.7, 95% CI 2.0-339.7, p = .03), while a history of prior VTE was also associated with increased risk (OR 26.0, 95% CI 0.9-796.8, p = .03). These findings may reflect increased venous stasis associated with prolonged immobility, prone positioning, and surgical manipulation in spinal procedures, while prior VTE represents an established risk factor for recurrent thromboembolism. However, these estimates should be interpreted cautiously because of the small number of events and extremely wide CIs. The multivariable regression estimates are therefore likely to be unstable and should be considered hypothesis-generating rather than precise estimates of independent risk.

The safety findings are reassuring but do not establish the safety of chemoprophylaxis. Surgical site bleeding, wound complications, and return to the operating room occurred at very low frequencies, and no statistically significant association with chemoprophylaxis was identified. However, the small number of bleeding events substantially limits the ability of this study to detect uncommon but clinically important hemorrhagic complications. The absence of a statistically significant increase in bleeding should therefore be interpreted as an absence of detected association rather than evidence that chemoprophylaxis is definitively safe.

The potential benefit of chemoprophylaxis must be weighed against the consequences of postoperative hemorrhage in neurosurgical patients. Hamilton et al. reported an absolute risk reduction of 9.1% for DVT with heparin prophylaxis, with an absolute risk increase of 0.7% for intracerebral hemorrhage and 2.8% for minor bleeding [7]. Ganau et al. similarly concluded that heparin prophylaxis carries a risk of postoperative hemorrhagic complications, with reported rates ranging from 2% to 4% [8]. The low rate of bleeding complications observed in our cohort may reflect institutional practices, including initiation of prophylaxis after adequate hemostasis was established and avoidance of prophylaxis in patients considered to be at high bleeding risk. However, because the decision to administer prophylaxis was not randomized and the reasons for withholding prophylaxis were not consistently documented, these practices may also contribute to selection bias and confounding by indication.

Several limitations should be considered when interpreting these findings. First, the retrospective, single-center design introduces the potential for selection bias, unmeasured confounding, and differences in baseline risk between patients who received and did not receive chemoprophylaxis. Confounding by indication is particularly important because patients at higher perceived risk for VTE may have been preferentially treated. Second, the study included only seven symptomatic VTE events, resulting in limited statistical power and wide confidence intervals. The low event rate makes the multivariable regression estimates unstable and limits the ability of the model to reliably determine independent predictors or estimate the treatment effect of chemoprophylaxis. Third, only symptomatic VTE events were evaluated, likely underestimating the overall incidence of thromboembolic disease compared with studies using routine screening ultrasonography.

Additional limitations include the heterogeneity of cranial and spinal procedures, the inclusion of both elective and urgent cases, and the requirement for a hospital stay of at least five days. These factors may limit generalizability to the broader neurosurgical population. Furthermore, information regarding the specific chemoprophylactic agent, duration of prophylaxis, and use of mechanical prophylaxis was limited. Although the timing of chemoprophylaxis initiation was not significantly associated with VTE in the subgroup analysis, the small number of events limits the ability to draw firm conclusions regarding the optimal timing of prophylaxis.

Despite these limitations, this study provides clinically relevant data regarding symptomatic VTE and postoperative complications in a heterogeneous neurosurgical population. The findings suggest that spinal surgery and prior VTE may identify patients at increased risk for symptomatic VTE and may support individualized risk stratification when determining prophylactic strategies. However, the study does not establish that chemoprophylaxis is ineffective or definitively safe. Rather, the results indicate that no statistically significant association between chemoprophylaxis and symptomatic VTE was detected in this cohort, while bleeding and wound complications were uncommon.

## Conclusions

In this retrospective cohort of postoperative neurosurgical patients, DVT chemoprophylaxis was not significantly associated with symptomatic VTE. The higher observed VTE rate among patients receiving chemoprophylaxis should not be interpreted as evidence of treatment failure given the potential for confounding by indication and the small number of outcome events. Bleeding and wound complications were uncommon, but the low number of events limits conclusions regarding the safety of chemoprophylaxis. Spinal surgery and prior VTE were associated with increased symptomatic VTE risk and may help identify patients who warrant individualized prophylactic strategies. Prospective multicenter studies with standardized prophylaxis protocols and larger sample sizes are needed to better define the efficacy, safety, and optimal timing of chemoprophylaxis in neurosurgical patients.

## References

[1] Borde TD, Prasad C, Arimappamagan A, Srinivas D, Somanna S (2017). Incidence of deep venous thrombosis in patients undergoing elective neurosurgery — a prospective cohort based study. Neurol India.

[2] Natsumeda M, Uzuka T, Watanabe J (2018). High incidence of deep vein thrombosis in the perioperative period of neurosurgical patients. World Neurosurg.

[3] Rethinasamy R, Alias A, Kandasamy R, Raffiq A, Looi MC, Hillda T (2019). Deep vein thrombosis and the neurosurgical patient. Malays J Med Sci.

[4] Henwood PC, Kennedy TM, Thomson L, Galanis T, Tzanis GL, Merli GJ, Kraft WK (2011). The incidence of deep vein thrombosis detected by routine surveillance ultrasound in neurosurgery patients receiving dual modality prophylaxis. J Thromb Thrombolysis.

[5] Khaldi A, Helo N, Schneck MJ, Origitano TC (2011). Venous thromboembolism: deep venous thrombosis and pulmonary embolism in a neurosurgical population. J Neurosurg.

[6] Nurmohamed MT, van Riel AM, Henkens CM (1996). Low molecular weight heparin and compression stockings in the prevention of venous thromboembolism in neurosurgery. Thromb Haemost.

[7] Hamilton MG, Yee WH, Hull RD, Ghali WA (2011). Venous thromboembolism prophylaxis in patients undergoing cranial neurosurgery: a systematic review and meta-analysis. Neurosurgery.

[8] Ganau M, Prisco L, Cebula H (2017). Risk of deep vein thrombosis in neurosurgery: state of the art on prophylaxis protocols and best clinical practices. J Clin Neurosci.

[9] Dickinson LD, Miller LD, Patel CP, Gupta SK (1998). Enoxaparin increases the incidence of postoperative intracranial hemorrhage when initiated preoperatively for deep venous thrombosis prophylaxis in patients with brain tumors. Neurosurgery.

[10] Collen JF, Jackson JL, Shorr AF, Moores LK (2008). Prevention of venous thromboembolism in neurosurgery: a metaanalysis. Chest.

[11] Agnelli G, Piovella F, Buoncristiani P (1998). Enoxaparin plus compression stockings compared with compression stockings alone in the prevention of venous thromboembolism after elective neurosurgery. N Engl J Med.

[12] Alshehri N, Cote DJ, Hulou MM, Alghamdi A, Alshahrani A, Mekary RA, Smith TR (2016). Venous thromboembolism prophylaxis in brain tumor patients undergoing craniotomy: a meta-analysis. J Neurooncol.

[13] Anderson DR, Morgano GP, Bennett C (2019). American Society of Hematology 2019 guidelines for management of venous thromboembolism: prevention of venous thromboembolism in surgical hospitalized patients. Blood Adv.

[14] Gould MK, Garcia DA, Wren SM, Karanicolas PJ, Arcelus JI, Heit JA, Samama CM (2012). Prevention of VTE in nonorthopedic surgical patients: Antithrombotic Therapy and Prevention of Thrombosis, 9th ed: American College of Chest Physicians Evidence-Based Clinical Practice Guidelines. Chest.

[15] Byrne JP, Witiw CD, Schuster JM (2022). Association of venous thromboembolism prophylaxis after neurosurgical intervention for traumatic brain injury with thromboembolic complications, repeated neurosurgery, and mortality. JAMA Surg.

[16] Li Q, Yu Z, Chen X, Wang J, Jiang G (2016). Risk factors for deep venous thrombosis of lower limbs in postoperative neurosurgical patients. Pak J Med Sci.

